# Regulation of plant biotic interactions and abiotic stress responses by inositol polyphosphates

**DOI:** 10.3389/fpls.2022.944515

**Published:** 2022-08-11

**Authors:** Esther Riemer, Naga Jyothi Pullagurla, Ranjana Yadav, Priyanshi Rana, Henning J. Jessen, Marília Kamleitner, Gabriel Schaaf, Debabrata Laha

**Affiliations:** ^1^Departmentof Plant Nutrition, Institute of Crop Science and Resource Conservation, Rheinische Friedrich-Wilhelms-Universität Bonn, Bonn, Germany; ^2^Department of Biochemistry, Indian Institute of Science, Bengaluru, India; ^3^Department of Chemistry and Pharmacy & CIBSS – The Center of Biological Signaling Studies, Albert-Ludwigs University Freiburg, Freiburg, Germany

**Keywords:** inositol pyrophosphate, jasmonic acid signaling pathway, salicylic acid signaling pathway, plant biotic and abiotic stress responses, phosphate homeostasis, auxin response

## Abstract

Inositol pyrophosphates (PP-InsPs), derivatives of inositol hexakisphosphate (phytic acid, InsP_6_) or lower inositol polyphosphates, are energy-rich signaling molecules that have critical regulatory functions in eukaryotes. In plants, the biosynthesis and the cellular targets of these messengers are not fully understood. This is because, in part, plants do not possess canonical InsP_6_ kinases and are able to synthesize PP-InsP isomers that appear to be absent in yeast or mammalian cells. This review will shed light on recent discoveries in the biosynthesis of these enigmatic messengers and on how they regulate important physiological processes in response to abiotic and biotic stresses in plants.

## Introduction

Inositol phosphates (InsPs) belong to the multifaceted family of signaling molecules that control a plethora of physiological processes across the eukaryote landscape (Shears, 2015; Thota and Bhandari, 2015; Laha et al., 2021a). These molecules are based on a six-carbon ring structure, *cis*-1,2,3,5-*trans*-4,6-cyclohexanehexol, commonly referred to as *myo*-inositol (Shears, 2015). Combinatorial phosphorylation of the *myo*-inositol ring could generate a large array of InsP species, of which only a few were identified in cell extracts (Shears, 2015). The physiological functions of most of these InsP species are largely unexplored. Almost 40 years ago, inositol 1,4,5-trisphosphate [Ins(1,4,5)P_3_] was shown for the first time to act as a second messenger, by acting as a calcium release factor that stimulates its specific receptor/Ca^2+^-permeable ion channel on endomembranes in pancreatic acinar cells (Streb et al., 1983; Irvine, 2003). In land plants, changes in intracellular InsP_3_ levels were shown to be responsive to various factors, such as root gravitropism, heat shock signal transduction, mechanical wounding, osmotic stress, pollen dormancy and blue light perception (Knight et al., 1997; Liu et al., 2006; Chen et al., 2008; Mosblech et al., 2008; Wang et al., 2009, 2012). These responses to InsP_3_ were assumed to be mediated by cytosolic Ca^2+^, as several studies showed that either treatment with caged photoactivatable compounds to release cytosolic InsP_3_ or direct InsP_3_ microinjection resulted in a transient increase in cytosolic Ca^2+^ (Blatt et al., 1990; Gilroy et al., 1990; Allen and Sanders, 1994; Tucker and Boss, 1996; Monteiro et al., 2005).

Additionally, it was shown that tomato plants expressing the human type I inositol polyphosphate 5-phosphatase (InsP5-ptase), a key enzyme in the phosphoinositide pathway, were more tolerant to drought and light stress, a phenotype that was suggested to be caused by the decrease of InsP_3_ detected in those plants (Khodakovskaya et al., 2010; Alimohammadi et al., 2012). Notably, even though InsP_1_, InsP_2_, InsP_3_, and InsP_4_ levels were shown to be decreased in InsP5-ptase-expressing plants, the role of other InsP species including PP-InsPs were not considered in these studies.

Importantly, genomes of flowering plants do not encode homologs of mammalian InsP_3_ receptors, which appear to have been lost during the course of evolution (Krinke et al., 2007; Wheeler and Brownlee, 2008; Munnik and Testerink, 2009; Munnik and Vermeer, 2010; Munnik and Nielsen, 2011; Zhang et al., 2018). Therefore, the role of Ins(1,4,5)P_3_ in plants remains unresolved. InsP_6_, also known as *myo*-inositol 1,2,3,4,5,6 hexakisphosphate, phytic acid or phytate, is the most abundant form of InsPs in eukaryotes, with concentrations in the range of 10–100 μM in animal and yeast cells, and up to 500 μM in slime molds (Wundenberg and Mayr, 2012; Pisani et al., 2014). InsP_6_ is the fully phosphorylated version of *myo*-inositol and serves as a phosphate (P_i_) storage molecule during seed development. In this process, InsP_6_ accumulates in storage microbodies in the form of mixed salts with cations, such as zinc, calcium, iron, potassium, magnesium and manganese (Raboy and Gerbasi, 1996; Otegui et al., 2002; Secco et al., 2017). The storage protein bodies are then degraded during seed germination, leading to the rapid hydrolysis of InsP_6_ by phytases to provide nutrients to the developing seedling (Raboy and Gerbasi, 1996; Loewus and Murthy, 2000). Due to its strong affinity toward different mineral cations, InsP_6_ is considered an antinutrient for humans and non-ruminant animals (McCance and Widdowson, 1942; Halsted et al., 1972). Since non-ruminant animals (e.g., pigs and poultry) lack phytases in their digestive tracts, excrements derived from phytate-rich diet contain phytate-bound phosphorus, which is often released in open water bodies, leading to eutrophication and environmental pollution (Rockström et al., 2009; Raboy, 2020).

InsP_6_ also serves as an important signaling molecule, directly or indirectly as a precursor of “di/pyro-phosphate”-containing inositol polyphosphates, commonly referred to as inositol pyrophosphates (PP-InsPs). These energy-rich InsP species are ubiquitous in eukaryotes, with InsP_7_ and InsP_8_ representing the most well-characterized species (Wilson et al., 2013; Shears, 2015). In plants, PP-InsPs control a range of important biological functions, including immune responses, hormone perception, and phosphate homeostasis (Zhang et al., 2007; Jadav et al., 2013; Laha et al., 2015, 2016, 2020; Jung et al., 2018; Kuo et al., 2018; Dong et al., 2019; Zhu et al., 2019; Gulabani et al., 2021; Land et al., 2021; Riemer et al., 2021).

**FIGURE 1:**
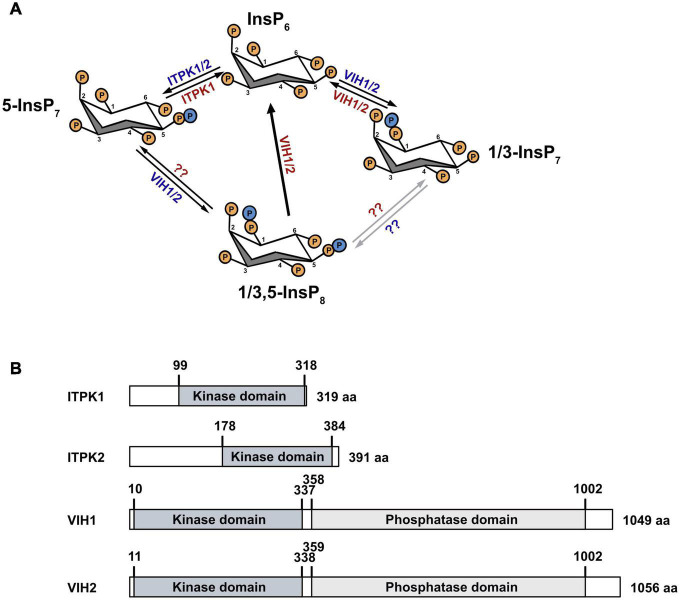
Inositol pyrophosphate biosynthesis pathway in plants and protein architecture of kinases. **(A)** ITPK1/2 and VIH1/2 phosphorylate InsP6 to generate 5-InsP7 and 1-InsP7, respectively. Further, VIH1/2 use 5-InsP7 as substrate to generate InsP8. The isomer identity of InsP8 remains unresolved but presumably represents the 1,5 and/or the 3,5-InsP8 isomer. VIH1/2 are also able to dephosphorylate 1/3,5-InsP8 and 1/3-InsP7 to InsP6. At low adenylate charge, the ITPK1 kinase domain also catalyzes the reverse reaction from 5-InsP7 to InsP6 in the presence of ADP to locally generate ATP. The gray arrows and question marks denote alternative routes of 1/3-InsP7 and 1/3,5-InsP8 synthesis, and the responsible enzymes, respectively. **(B)** Schematic representation of ITPK1, ITPK2, VIH1 and VIH2 architectures. Kinase domains are shown in dark gray, phosphatase domains in light gray.

The metabolic pathways leading to the production of PP-InsPs are well-established in metazoan and yeast. In these organisms, mammalian IP6K/yeast Kcs1-type kinases catalyze the phosphorylation of InsP_6_ or 1-InsP_7_ at the 5 position, resulting in the generation of 5-InsP_7_ or 1,5-InsP_8_, respectively (Saiardi et al., 1999; Draskovic et al., 2008). Furthermore, mammalian PPIP5K/yeast Vip1 kinases phosphorylate the 1 position of InsP_6_ and 5-InsP_7_ to generate 1-InsP_7_ and 1,5-InsP_8_, respectively (Mulugu et al., 2007; Lin et al., 2009; Wang et al., 2011; Zhu et al., 2019). The PP-InsP biosynthetic pathway is partially conserved in plants. For instance, while the Arabidopsis genome encodes Vip1 isoforms, genes encoding Kcs1-type kinase(s) could not be identified yet. However, recent studies have demonstrated that the *Arabidopsis thaliana* kinases ITPK1 and ITPK2 phosphorylate InsP_6_, which is first generated by the phosphorylation of InsP_5_ [2-OH] by IPK1, to synthesize 5-InsP_7_ *in vitro* (Adepoju et al., 2019; Laha et al., 2019; Whitfield et al., 2020) and *in planta* (Parvin Laha et al., 2020; Riemer et al., 2021). These proteins belong to the family of ATP-grasp fold proteins with the capability to bind ATP in a cleft between the β sheet toward the central and C-terminal domain (Miller et al., 2005; Josefsen et al., 2007). Notably, their homologs ITPK3 and ITPK4 do not appear to phosphorylate InsP_6_ *in vitro* or *in vivo* (Laha et al., 2019). The Arabidopsis Vip1 isoforms VIH1 and VIH2 harbor both an N-terminal ATP-grasp kinase domain, as well as a C-terminal phosphatase-like domain and are responsible for InsP_8_ production *in planta* (Figure 1A; Laha et al., 2015; Zhu et al., 2019).

The recent establishment of novel methods for InsP analyses led to the emergence of several plant PP-InsP studies, which have been instrumental to establish PP-InsP as novel signaling molecules in plants. Remarkably, to date it is still challenging to separate different PP-InsP isomers. This leads to the open question, whether the enantiomers 1-InsP_7_ or 3-InsP_7_ and 1,5-InsP_8_ or 3,5-InsP_8_ are the main isomers in plants or, if both exist, which of them is the most abundant.

Inositol pyrophosphates play a crucial role in the adaption to several stress responses in plants. Previous work has demonstrated the relevance of InsP_7_ and InsP_8_ in responses to hormones such as auxin, salicylic acid or jasmonate (Laha et al., 2015, 2016; Parvin Laha et al., 2020; Gulabani et al., 2021).

In addition, P_i_ homeostasis in plants was shown to be regulated by kinases involved in InsP synthesis (Kuo et al., 2014, 2018), most likely due to their contribution to the synthesis of InsP_8_, which serves as a proxy for P_i_ (Dong et al., 2019; Zhu et al., 2019; Riemer et al., 2021). Interestingly, certain bacterial plant pathogens interfere with plant InsP_6_- and potentially PP-InsP-dependent hormone signaling by injecting XopH-like type III effectors that function as 1-phytases (Blüher et al., 2017). However, it is still unclear how this modulation of the host’s InsP and PP-InsP status benefits the pathogen (Blüher et al., 2017). Beyond that, recent studies demonstrated a link between pathogen defense and P_i_ starvation, displaying InsPs and PP-InsPs as crosstalk mediators of abiotic and biotic stresses (Gulabani et al., 2021).

In this review, we present in detail the latest findings of the roles of these phosphate-rich molecules in regulating different biotic and abiotic responses in plants.

## Enzymatic activity of PP-InsP kinases

The function of InsP and PP-InsP kinases is not only limited to the generation of higher inositol pyrophosphates, as they can also shift their activity from PP-InsP synthases to ATP synthases in response to different ATP ratios (Voglmaier et al., 1996; Gu et al., 2017; Zhu et al., 2019; Riemer et al., 2021). It was already shown that mammalian IP6K kinases can transfer a phosphate group from InsP_7_ to ADP to generate ATP (Voglmaier et al., 1996). Furthermore, both mammalian and yeast IP6K/Kcs1 activities react to changes in cellular ATP levels with respect to the generation of 5-InsP_7_ (Saiardi et al., 1999; Gu et al., 2017).

In contrast to IP6K/Kcs1, which harbor only a kinase domain, mammalian and yeast PPIP5K/Vip1 harbor both an N-terminal kinase domain and a C-terminal phosphatase domain in the same protein, enabling them to act as bifunctional enzymes (Fridy et al., 2007; Mulugu et al., 2007; Wang et al., 2015; Zhu et al., 2019). As mentioned above, Arabidopsis VIH1 and VIH2 also possess an N-terminal kinase and a C-terminal phosphatase domain (Figure 1B). Similarly to the mammalian IP6K (Voglmaier et al., 1996), Arabidopsis ITPK1 does not only transfer phosphates to inositol polyphosphates but also acts as ATP synthase under varying ATP/ADP ratios or P_i_ concentrations (Figure 1B). The enzyme has a high K_*M*_ of 520 μM for ATP and shifts its activity from kinase to ATP synthase at low adenylate energy charges by transferring the β-phosphate from 5-InsP_7_ to generate ATP from ADP (Riemer et al., 2021). In addition, ITPK1 exclusively uses 5-InsP_7_ and no other InsP_7_ isomer as a substrate for this ADP phosphotransferase activity *in vitro*, in agreement with a high substrate specificity (Riemer et al., 2021). Besides, ITPK1 was shown to act as an InsP(3,4,5,6)_4_ 1-kinase/InsP_5_ [2-OH] 1-phosphotranferase to generate ATP from ADP *in vitro* (Whitfield et al., 2020).

Taken together, plant PP-InsP kinases catalyze both the generation and the removal of PP-InsPs. This raises the hypothesis that the enzymes might modulate energy reserves by shifting their activities, for instance, in response to environmental changes, such as phosphorus limitation or sufficiency (Saiardi et al., 1999; Riemer et al., 2021).

## Discovery of new PP-InsP_4_ and InsP_7_ isomers in plants

The detection and quantification of plant PP-InsP species is challenging due to their low abundance, as well as the presence of high amounts of acid phosphatases in plant extracts, which leads to rapid degradation of PP-InsPs (Laha et al., 2021b). Until recently, Strong Anion Exchange High Performance Liquid Chromatography (SAX-HPLC) and Polyacrylamide Gel Electrophoresis (PAGE) were the most common methods used to analyze InsPs and PP-InsPs (Azevedo and Saiardi, 2006; Pisani et al., 2014). Owing to its easy set-up and low costs, PAGE is still widely employed to resolve higher inositol polyphosphates. The drawback of both of the above-mentioned methods is the inability to separate PP-InsP isomers. The first clarification of isomer identity of a particular PP-InsP species in plant tissue was possible *via* two-dimensional nuclear magnetic resonance spectroscopy (NMR), by taking advantage of an Arabidopsis *mrp5* mutant (Laha et al., 2019). This mutant is defective in vacuolar loading of InsP_6_, leading to elevated PP-InsP cyto/nucleoplasmic levels (Nagy et al., 2009; Desai et al., 2014; Laha et al., 2019; Riemer et al., 2021). NMR analyses of *mrp5* seed extracts and comparison with synthetic references demonstrated that 5-InsP_7_ is the major PP-InsP species present in *mrp5* seeds (Laha et al., 2019).

Coupling of the two powerful tools “capillary electrophoresis” and “electrospray ionization mass spectrometry” (CE-ESI-MS) has enabled new insights into the abundance of InsP and PP-InsP isomers in mammalian cells, yeast, amoeba and plants (Qiu et al., 2020). Due to its high tolerance of complex sample matrices, the combined CE-ESI-MS enables separation of highly charged metabolites with compelling sensitivity. By employing this technique, the generation of 1/3-InsP_7_ and 5-InsP_7_ by VIH2 and ITPK1, respectively, was finally confirmed *in planta* (Riemer et al., 2021). Notably, this work also revealed for the first time the presence of 4/6-InsP_7_ in plants. In Arabidopsis, this InsP_7_ isomer was found to be more prominent than 5-InsP_7_ and 1/3-InsP_7_. However, in contrast to 1/3-InsP_7_ and 5-InsP_7_, the new isomer is less responsive to P_i_ deplete and replete conditions (Riemer et al., 2021) and the function(s) of 4/6-InsP_7_ and the potential kinase(s) that generate this new isomer in plants are still unknown.

Notably, not only InsP_6_ is converted to higher PP-InsPs, but also isomers of pentakisphosphates (InsP_5_) can serve as precursors for the generation of 5-diphosphoinositol tetrakisphosphate (5PP-InsP_4_) in yeast and mammalian cells (Wang et al., 2018). For instance, Saiardi et al. (2000) demonstrated that the yeast InsP_6_ kinase Kcs1 can generate PP-InsP_4_ from InsP_5_ [2-OH] *in vitro*. Interestingly, the affinity of Kcs1 for InsP_5_ was shown to be threefold higher (mean *K*_*M*_ = 1.2 μM) than for InsP_6_ (mean *K*_*M*_ = 3.3 μM). The mammalian IP6K1 also phosphorylates InsP_5_ [2-OH] to PP-InsP_4_, but in this case with similar affinities for InsP_5_ and InsP_6_ phosphorylation (Saiardi et al., 2000). While InsP_5_ levels in yeast are low and therefore probably do not represent the main Kcs1 substrate *in vivo*, this might be different in mammalian cells, where InsP_5_ [2-OH] and InsP_6_ levels are similar and represent physiologically relevant substrates of IP6K1 (Saiardi et al., 2000).

A recent study reported the identification of a novel PP-InsP_4_ isomer that does not co-migrate with a synthetic 5PP-InsP_4_ standard, suggesting a distinct structural identity as compared to PP- InsP_4_ isomers identified in yeast and mammalian cells (Riemer et al., 2021). CE-MS and PAGE data show that this plant PP-InsP_4_ isomer increases under P_i_-starvation, as well as under P_i_-resupply conditions, and is not detectable in nutrient-repleted plant roots. Interestingly, in roots of *itpk1* loss-of-function mutants, this novel PP-InsP isomer seems to be less abundant, suggesting that ITPK1 might catalyze the generation of PP-InsP_4_ *in planta* (Riemer et al., 2021). Interestingly, ITPK1 catalyzes the generation of PP-InsP_4_ from InsP_5_ [6-OH] *in vitro* (Whitfield et al., 2020) but it remains to be shown whether plants possess the InsP_5_ [6-OH] isomer and whether the ITPK1-dependent PP-InsP_4_ derives from it.

The finding that other PP-InsP species than 1/3-InsP_7_ or 5-InsP_7_ were detected in plant extracts unveil an unexplored diversity of inositol pyrophosphates in plants. Also the involvement of putative unknown kinases responsible for the production of additional isomers in environmental responses still have to be investigated.

## Inositol pyrophosphate kinases and their role in the adaption of plants to biotic and abiotic stress responses

### Inositol pyrophosphate kinases are involved in salicylic acid-dependent immunity

The plant hormone salicylic acid (SA) regulates several processes like flower induction, stomatal closure and heat production mediated by alternative respiration in flowers (Rai et al., 1986; Raskin, 1992). Besides, SA is known to play a crucial role in defense mechanisms against bacteria, fungi, viruses and insects (Raskin et al., 1989; Chaerle et al., 1999; Martínez et al., 2004; Zarate et al., 2007; Kim and Hwang, 2014; Hao et al., 2018). Plant immune responses include the so-called PAMP-triggered immunity (PTI), characterized by the recognition of pathogen-associated molecular patterns (PAMPs, e.g., the bacterial peptide flagellin 22, or flg22), which triggers ion fluxes, ROS production and a series of signaling cascades that ultimately lead to local or systemic responses to restrict pathogen invasion (Seybold et al., 2014). Besides PTI, plants count on a second layer of protection, the effector triggered immunity (ETI), in which plants recognize effector proteins secreted by the pathogen. The ETI usually triggers fast defense reactions, such as hypersensitive response (HR), to promptly restrict pathogen colonization (Dodds and Rathjen, 2010).

Both PTI and ETI are modulated by SA, which is also key for the establishment of systemic acquired resistance (SAR), an additional layer of defense that protects plants from subsequent pathogen attacks (Hõrak, 2020). For instance, SA activates, *via* the regulatory protein NPR1, expression of several pathogenesis-related (PR) genes, which encode different types of proteins with antimicrobial properties (Van Loon et al., 2006; Hõrak, 2020).

A defined role of PP-InsPs in SA-signaling is still unclear. This is because studies showing an involvement of InsPs and PP-InsPs in SA-dependent immunity have in part contradictory outcomes. Arabidopsis mutants disrupted in InsP_6_ biosynthesis, for instance, showed increased susceptibility to bacterial, fungal and viral infections (Murphy et al., 2008; Poon et al., 2020), as well as to cyst nematode infestation (Jain, 2015). In fact, the Arabidopsis *ips2* and *ipk1* mutants defective in the activities of enzymes for the first and last steps in InsP_6_ biosynthesis, respectively, were similarly susceptible to microbial pathogens than *NahG*-transgenic lines and to *sid2* mutants, both of which are unable to accumulate normal levels of SA (Murphy et al., 2008). The SA contents in *ips2* and *ipk1*, however, did not differ from those of wild-type plants, and also increased, similarly to wild-type, after challenge with *Pseudomonas syringae* pv. *tomato* (*Pst*) DC3000 *AvrB* (Murphy et al., 2008). These results indicate that the enhanced susceptibility of *ips2* and *ipk1* is not related to low SA levels, but could be caused by the disruption of InsP_6_ biosynthesis (Murphy et al., 2008). Further studies of the *ipk1* mutant and of loss-of-function mutants of another *IPS* isoform (*IPS3*) confirmed an involvement of InsP_6_ in basal pathogen responses (Poon et al., 2020). While displaying a higher susceptibility to *Pst* than wild-type plants, when the *ips2*, *ips3*, and *ipk1* mutants were assessed for SAR acquirement, no impairment was detected. Besides, all mutants except *ipk1* presented flg22-induced resistance to *Pst*, indicating that PTI was inhibited in *ipk1* only (Poon et al., 2020). In this case, however, disruption of InsP_6_ synthesis in *ipk1* did not affect typical responses to flg22, such as Ca^2+^ influx, oxidative burst, root growth inhibition and activation of PAMP-triggered genes. Taken together, these data suggest that InsP_6_ biosynthesis is important for maintaining basal resistance against various pathogens, contributing to defense mechanisms different from canonical PTI (Murphy et al., 2008; Poon et al., 2020).

In contrast to findings presented by those previous studies, a recent analysis of *ipk1*, *itpk1*, and *vih2* mutants revealed that these enzymes act as negative regulators of SA-dependent immunity (Gulabani et al., 2021). Mutant plants, in which either InsP_7_ or InsP_8_ levels are impaired (Stevenson-Paulik et al., 2005; Sweetman et al., 2007; Desai et al., 2014; Laha et al., 2015; Kuo et al., 2018; Riemer et al., 2021), were significantly more resistant to bacterial infection by *Pst* in comparison to wild-type (Gulabani et al., 2021). Such a response was associated with an apparent constitutive activation of defenses observed in these plants. For instance, they showed a strong upregulation of SA biosynthesis genes, such as *SID2/ICS1*, and higher levels of free or glycolsyl moiety-conjugated SA (SAG) than wild-type plants. Along with these findings, an increase in the expression of *PR1* and *PR2*, together with an accumulation of the respective proteins was observed. Also protein levels of ENHANCED DISEASE SUSCEPTIBILITY 1 (EDS1) and SUPPRESSOR OF nrp1-1 CONSTITUTIVE 1 (SNC1), both of which are required for basal defenses, were higher in these mutants than in wild-type plants, probably due to their elevated SA levels (Gulabani et al., 2021).

Although previous studies highlighted the importance of InsP_6_ in maintaining basal defenses against bacteria (Murphy et al., 2008; Ma et al., 2017; Poon et al., 2020), a set of mutants reduced in phytic acid levels, such as *mik-1*, *ipk2*β, or *itpk4*, displayed comparable PTI to wild-type (Gulabani et al., 2021). These findings suggest that InsP_6_ is not directly involved in triggering plant defenses but point toward a role of higher inositol pyrophosphates in regulating basal immunity (Gulabani et al., 2021).

Currently, it remains unclear whether InsP_7_ or InsP_8_ is the main player in SA-mediated defense. Both molecules might act indirectly by the regulation of an antagonistic crosstalk between auxin-SA and jasmonic-acid (JA)-SA, respectively. As described in details in section “As described in the section 5.5,” of this review, 5-InsP_7_ was proposed to regulate auxin signaling by acting as a co-ligand of the ASK1-TIR1-Aux/IAA auxin receptor complex (Parvin Laha et al., 2020), and exogenous application of auxin enhances the *Pst* infestation by interfering with SA-defenses (Navarro et al., 2006; Wang et al., 2007). Therefore, disruption of auxin signaling in *itpk1* mutants might enhance basal immunity (Gulabani et al., 2021). On the other hand, the antagonism between JA-SA crosstalk in plants is well-described and even pathogens have the capability to secrete hormone-mimicking effectors to hijack host defense mechanisms (Zheng et al., 2012; Caarls et al., 2015). For instance, coronatine, a *Pst*-produced phytotoxin that mimics JA, triggers virulence by downregulating SA-dependent defenses in plants (Brooks et al., 2005; Zheng et al., 2012). Furthermore, several studies indicate that endogenous SA is antagonistic to JA-dependent defense mechanisms in plants, leading to a prioritized SA-driven resistance over JA-regulated defense (reviewed in Pieterse et al., 2012). Along these lines, *ipk1* plants primed with injection of air or water to mimic wounding were less susceptible to *Pst* than corresponding wild-type plants that were primed in the same way (Poon et al., 2020). It remains unclear which *ipk1*-dependent inositol phosphate species might be responsible for this phenotype, as *ipk1* mutants are defective in InsP_6_, InsP_7_, and InsP_8_ synthesis (Laha et al., 2015; Gulabani et al., 2021).

Taken together, elevated SA levels and expression of PTI-responsive genes in *ipk1*, *itpk1* and *vih2* might be related to disrupted JA signaling by low PP-InsP levels, causing enhanced SA-defense mechanisms (Gulabani et al., 2021). Further research is needed to unveil the involvement of specific PP-InsPs and other InsP species in regulating plant SA-dependent immunity.

### The role of inositol pyrophosphates in jasmonate perception

Jasmonic acid and its derivates, collectively known as jasmonates (JA), play a crucial role in regulating plant development and defense against several necrotrophs and herbivores (Wasternack and Hause, 2013). In response to wounding or herbivory insects, the level of the bioactive JA conjugate jasmonic isoleucine (JA-Ile) is elevated (Fonseca et al., 2009; Koo et al., 2009), which in turn binds to the Coronatine Insensitive 1 (COI1) protein (Feys et al., 1994; Xie et al., 1998; Xu et al., 2002; Katsir et al., 2008), the F-box component of the SCF ubiquitin E3 ligase complex (Devoto et al., 2002). Binding of JA-Ile to COI1 recruits the Jasmonate ZIM Domain (JAZ) transcriptional repressor, which subsequently undergoes polyubiquitylation and SCF^*COI*1^-mediated proteasome degradation (Chini et al., 2007; Thines et al., 2007; Yan et al., 2007). JAZ degradation then de-represses MYC2 and other transcription factors and consequently triggers the expression of JA-dependent genes (Boter et al., 2004; Browse, 2009).

Crystallization of the insect-purified auxin receptor TIR1/IAA complex that contained insect-derived InsP_6_ provided important information to better understand phytohormone-mediated signaling in plants (Tan et al., 2007). Nano-electrospray mass spectroscopy of the ASK1-COI1 complex that was purified from an insect cell line ectopically expressing the Arabidopsis ASK1-COI1 complex indeed revealed the existence of a molecule whose molar mass corresponded to InsP_5_ (Sheard et al., 2010). A multistep purification strategy followed by ^1^H NMR analysis and Total Correlation Spectroscopy (TOCSY) allowed to identify this ligand as either InsP_5_ [1-OH] or InsP_5_ [3-OH] (Sheard et al., 2010). In the crystal structure of the ASK1-COI1 complex, the presence of strong electron densities congregating in the core of the solenoid structure likely represents individual phosphates that replaced the insect-derived InsP5 ligand, probably due to high concentrations of ammonium phosphate, used as a precipitant during crystallization (Sheard et al., 2010). To further evaluate the functional role of InsPs in ASK1-COI1-JAZ1 co-receptor complex, a ligand-binding reconstitution assay revealed that both Ins(1,4,5,6)P_4_ and InsP_5_ [3-OH] can strongly induce ASK1-COI1-JAZ co-receptor complex formation *in vitro*, whereas InsP_6_ appeared to be less effective (Sheard et al., 2010). However, it is still unclear whether plants contain InsP_5_ [3-OH], its enantiomer InsP_5_ [1-OH] or both isomers.

Several studies pointed to an involvement of InsPs in wound response, as well as disease resistance in plants (Mosblech et al., 2008; Murphy et al., 2008). Arabidopsis plants heterologously expressing human inositol phosphate 5-phosphatase exhibit reduced levels of InsP_3_ (Perera et al., 2006; Hung et al., 2014) and are found to be susceptible to the cabbage moth *Plutella xylostella* (Mosblech et al., 2008). As previously mentioned, Murphy et al. (2008) also showed that Arabidopsis *ipk1* and *ics2* mutant plants with defects the in production of InsP_6_ are compromised in plant defense against bacterial (*Pseudomonas syringae*), viral (*Tobacco Mosaic Virus*), and necrotrophic fungal (*Botrytis cinerea*) pathogens.

To gain insights into the functional role of InsPs in JA signaling, mutant lines defective in putative inositol phosphate binding residues of COI1 were analyzed (Mosblech et al., 2011). Yeast two-hybrid (Y2H) analysis of the COI1 mutant variants revealed reduced interaction with JAZ proteins in presence of the JA analog coronatine, which suggests that InsP binding to the receptor complex might be important (Mosblech et al., 2011). Supporting this statement, Y2H studies using mutant lines defective in InsP biosynthesis revealed an enhanced COI1/JAZ interaction in the *ipk1*Δ yeast strain, which has high levels of InsP_5_ [2-OH] (Mosblech et al., 2011).

Although one cannot simply compare the yeast *ipk1*Δ mutant with Arabidopsis *ipk1-1* lines, as yeast *ipk1* strains show elevated levels of a specific PP-InsP_4_ isomer that cannot be detected in Arabidopsis (Saiardi et al., 2002; Laha et al., 2015; Riemer et al., 2021), these studies suggest a potential role of InsPs in regulating JA responses in plants. The enhanced interaction of COI1 and JAZ in *ipk1*Δ yeast strain could also be explained by the high levels of PP-InsP_4_. Additionally, Arabidopsis *ipk1-1* plants are not only defective in InsP_6_ but are also severely compromised in InsP_7_ and InsP_8_ (Laha et al., 2015). To further evaluate the potential role of PP-InsPs in JA-dependent responses, VIH2-deficient plants defective in InsP_8_ synthesis were investigated (Laha et al., 2015). The mutant plants had unchanged levels of InsP_5_ [2-OH], but were shown to be severely susceptible to the generalist herbivore *Mamestra brassicae* and the Brassicaceae specialist *Pieris rapae*. This suggests that VIH2-dependent InsP_8_ but not InsP_5_ [2-OH] is critical for defense against these insects (Laha et al., 2015). In addition, *vih2* mutants showed reduced expression of JA-dependent genes, despite an increase in JA. Therefore, the compromised resistance of Arabidopsis *vih2* mutants against herbivory insects might be explained by a defect in JA perception, and not by compromised JA production (Laha et al., 2015). Additionally, *in vitro* binding assays of ASK1-COI1-JAZ1-coronatine with different radiolabeled InsPs indicated that higher inositol polyphosphates, such as InsP_6_ and InsP_7_, are capable to bind to the JA-receptor complex with higher efficiency than lower InsPs (Laha et al., 2015, 2016). Unfortunately, plant-purified InsP_8_ was not included in these binding assays due to its low amounts in plants and its high susceptibility to acid phosphatases present in plant extracts (Laha et al., 2015).

Taken together, it has been proposed that coincidence detection of VIH2-dependent InsP_8_ and JA is important for plant defense against necrotrophic and herbivorous pathogens (Laha et al., 2015, 2016). While these studies provide some mechanistic insights into the role of InsP_8_ in JA responses, future work is needed to clarify the molecular basis of VIH2 functions. For instance, it has not been established whether the catalytic activity of VIH2 solely contributes to JA responses, or whether VIH2 regulates JA responses through a yet unidentified mechanism. It might be also interesting to learn whether the phosphatase domain of VIH2 contributes to JA-related defense responses.

The studies presented here led to the assumption that plants are able to use different InsPs to cope against several pathogens and herbivores (Mosblech et al., 2011; Laha et al., 2015). However, whether InsPs allow plants to differentially respond against pathogens and how this process takes place remains an interesting question. The precise mechanism by which the JA co-receptor complex discriminates and specifically binds to a particular InsP isomer is still unclear. Furthermore, the physiological relevance of various InsP isomers in the context of the JA signaling pathway is still unsolved. It would be interesting to explore the possibility that different InsPs could form a series of distinctive JA-co-receptor complexes, which would help plants to induce specific immune responses against distinct pathogens.

### The 1-phytase activity of *Xanthomonas* type III effector XopH

Several Gram-negative bacteria of the genus *Xanthomonas* cause diseases in different plant hosts, such as pepper, rice, wheat, tomato, citrus, cabbage, and banana, leading to substantial crop yield losses (Ryan et al., 2011; Jacques et al., 2016). A broad range of factors influence host specificity and pathogenicity. These include bacterial lipopolysaccharides, adhesins, transcription factors and TonB-dependent receptors, as well as the type III secretion system (T3SS) (Raetz and Whitfield, 2002; Ghosh, 2004; Blanvillain et al., 2007; Das et al., 2009; Büttner, 2016). The latter is responsible for the translocation of effector proteins into the plant cell cytosol (Büttner, 2016; Constantin et al., 2017; Newberry et al., 2019). The tomato and pepper pathogen *Xanthomonas campestris* pv. *vesicatoria* (Xcv) encodes more than 30 T3S effector proteins, which are generally designated as Xops (*Xanthomonas* outer proteins) and are known to cause characteristic bacterial spot disease symptoms (Thieme et al., 2005; Teper et al., 2015). In resistant plants, the effectors are recognized by immune receptors, often leading to HR at the infected area to suppress spreading of biotrophic pathogens from the site of infection (Goodman and Novacky, 1994; Mur et al., 2008). One member of the Xops effector family, XopH, depicts typical features of dual-specific protein phosphatases and can dephosphorylate the generic substrate p-nitrophenyl phosphate (pNPP) (Potnis et al., 2012).

Blüher et al. (2017) reported a novel phytate-degrading activity of XopH *in vitro* and *in planta*, which is assumed to account for the activation of HR in resistant plants. Using a novel NMR method coupled with spiking experiments, as well as biochemical studies with recombinant XopH, the authors identified XopH as a 1-phytase that cleaves the phosphate from the C1 hydroxy group of InsP_6_, resulting in the generation of InsP_5_ [1-OH] (Blüher et al., 2017). HPLC data of *S. cerevisiae* and *N. benthamiana* ectopically expressing XopH revealed a reduction of InsP_6_ and a strong accumulation of InsP_5_ [1/3-OH] also *in vivo* (Blüher et al., 2017). To confirm whether XopH executes 1-phytase activity *in planta*, the authors performed XopH digestion of InsP_5_ [1/3-OH] species purified from [3H]-*myo*-inositol-labeled transgenic *N. benthamiana* overexpressing *xopH*. The plant-purified InsP_5_ [1/3-OH] was resistant to XopH degradation and was not phosphorylated by plant enzymes, supporting the idea that this PP-InsP isomer is absent in plants and is more likely a product of XopH phytase activity (Blüher et al., 2017). Strikingly, the XopH-induced HR in pepper plants harboring the *Bs7* resistance (R) gene seems to be dependent on the effector’s phytase activity. This led to the assumption that Bs7 more likely recognizes the result of XopH activity, such as changes in inositol polyphosphate levels, but not the protein itself (Blüher et al., 2017). It was also observed that heterologous expression of XopH in *N. benthamiana* resulted in a strong reduction of InsP_7_ and InsP_8_, presumably interfering with InsP_7_- and InsP_8_-dependent hormone signaling. In agreement with this, qRT-PCR analysis of *N. benthamiana* leaves constitutively expressing *xopH* showed an induction of the JA marker genes *PR1b*, *PR4*, and *PI-II* after wounding, strengthening the involvement of the effector protein in JA signaling (Blüher et al., 2017). Since those genes are also responsive to ethylene (ET), a hormone acting synergistically to JA, it cannot be excluded that XopH also affects the ET pathway. Indeed, virus-induced gene silencing of *EIN2* and *EBF1*, which are the positive and negative regulators of the ET pathway, respectively, caused the suppression of *xopH*-induced upregulation of *PR4* and *PI-II* in *N. benthamiana* (Donnell et al., 1996; Adie et al., 2007; Zhu and Lee, 2015; Blüher et al., 2017).

It still remains unclear for what purpose Xanthomonas secretes XopH into the host cells. One possibility is that the XopH phytase activity might release phosphate from the plant tissue, which could enhance the nutritional status of the pathogen. A similar activity was observed for the phytase PhyA, which is secreted by the rice pathogen *X. oryzae* pv. *Oryzae*. It was suggested that this bacterial pathogen uses phytate as the sole phosphate source and that this activity contributes to its virulence (Chatterjee et al., 2003). In addition, XopH might also degrade higher inositol pyrophosphates and thereby influence hormone signaling pathways of the host, leading to manipulation of JA- or ET-mediated defense responses to the pathogen’s benefit (Blüher et al., 2017).

### Inositol pyrophosphates are involved in phosphate homeostasis

Phosphorus is an essential element and a key determinant for growth and development of all living organisms, as it composes essential molecules such as ATP, nucleic acids and phospholipids (Marschner, 1995). Plants take up phosphorus in the form of P_i_, which is highly immobile, chemically fixated, as well as unevenly distributed in soils, causing a very limited access of available P_i_ (Holford, 1997; Seidel et al., 2021). Plants respond to low P_i_ levels by metabolic changes such as an increase of sulfo- and galactolipids at the expense of phospholipids (Essigmann et al., 1998; Härtel et al., 2000) and by increasing RNA degradation to release phosphate for other cellular processes (Taylor et al., 1993; Bariola et al., 1994). Furthermore, P_i_-starved plants increase P_i_ acquisition *via* the production and secretion of phosphatases, exudation of organic acids, modification of root architecture and development of root hairs, as well as enhanced expression of P_i_ transporters (Karthikeyan et al., 2002; Mudge et al., 2002; Rausch and Bucher, 2002; Vance et al., 2003; Shin et al., 2004; Plaxton and Tran, 2011; Péret et al., 2011). These metabolic, morphological and transcriptional mechanisms belong to the so called phosphate starvation response (PSR), which is interrupted upon P_i_ replenishment (Vance et al., 2003; Chiou and Lin, 2011; Secco et al., 2013).

The majority of P_i_ starvation-induced (PSI) genes in plants is regulated by the MYB-CC transcription factor PHOSPHATE STARVATION RESPONSE REGULATOR 1 (PHR1) and its homolog PHR1-LIKE 1 (PHL1) (Rubio et al., 2001). PHR1 is expressed under P_i_-sufficient conditions and controls P_i_ signaling and homeostasis through binding as a dimer to an imperfect palindromic sequence (PHR1-binding sequence, or P1BS) present in the promoters of P_i_ starvation-induced genes (Rubio et al., 2001).

Recent studies showed that a class of stand-alone SPX (SYG1/Pho81/XPR1-domain containing protein 1) proteins negatively regulates the activity of PHR transcription factors by high affinity binding to PHRs under sufficient P_i_ supply (Puga et al., 2014). The formation of the SPX-PHR complex in turn prevents the binding of the transcription factors to the P1BS motifs, thereby repressing the expression of PSI genes. Under low P_i_ conditions, the binding affinity of SPX to the PHRs is decreased, leading to the activation of their transcriptional targets (Puga et al., 2014; Qi et al., 2017).

Structural studies of SPX domains from proteins of different organisms indicate that PP-InsPs bind to SPX domains on a conserved cluster of basic residues and regulate the activity of such proteins, as shown for an SPX-containing component of the Vacuolar Transporter Chaperone (VTC) complex that mediates polyphosphate synthesis in baker’s yeast (Wild et al., 2016). Similar conserved clusters of basic residues at the surface of the SPX N-terminus were also identified in plant SPX proteins (Wild et al., 2016).

Recently, Dong et al. (2019) demonstrated that InsP_8_ binds to the rice *Os*SPX1 domain with a Kd of approximately 5.7 μM *in vitro*. In addition, Co-IP results revealed that SPX1 is not able to interact with PHR1 under P_i_ starvation conditions but can be restored by adding 1 μM InsP_8_ (Dong et al., 2019). On the other hand, the SPX-PHR interaction cannot be restored by the addition of InsP_7_, corroborating the idea that InsP_8_ but not InsP_7_ acts as the key regulator of P_i_ starvation responses in plants (Dong et al., 2019).

Several *in vivo* studies confirmed the involvement of PP-InsPs in PSR in plants. Arabidopsis mutants defective in IPK1 activity exhibit a disturbed phosphate starvation phenotype (Kuo et al., 2014). This results in an increased P_i_ overaccumulation when grown under P_i_ sufficient conditions and P_i_ accumulation in response to increasing external P_i_ concentrations (Stevenson-Paulik et al., 2005; Kuo et al., 2014, 2018). In addition, the mutants displayed reduced levels of InsP_6_, InsP_7_, and InsP_8_ (Laha et al., 2015; Kuo et al., 2018; Land et al., 2021).

Under P_i_-replete conditions, the loss of *ITPK1* but not of *ITPK2* causes a robust overaccumulation of P_i_ similar to what was observed in *ipk1* plants, even though only a decrease in 5-InsP_7_ and not in InsP_8_ was observed in *itpk1* plants under such conditions (Riemer et al., 2021). In contrast, P_i_-starved *itpk1* plants that were resupplied with P_i_ displayed strong defects in both 5-InsP_7_ and InsP_8_ synthesis, again coinciding with a robust PSR phenotype (Riemer et al., 2021). An earlier study reported reduced InsP_8_ levels of *itpk1* plants, as revealed by PAGE analyses also under P_i_-replete conditions (Wang et al., 2021). The difference between the works of Riemer et al. (2021) and Wang et al. (2021) might be explained by different growth conditions employed by these two independent studies, including different P_i_-availabilities at the P_i_-replete condition.

While disruption of VIH1 and VIH2 did not cause any PSR phenotype, such as PSR gene expression and P_i_-accumulation under P_i_-replete conditions (Kuo et al., 2018; Land et al., 2021; Riemer et al., 2021), loss of VIH2 caused a mild PSR phenotype upon P_i_-resupply to P_i_-starved plants (Riemer et al., 2021). Importantly, *vih1 vih2* double mutants (in which the respective kinase domains are defective) are seedling lethal (Zhu et al., 2019).

**FIGURE 2:**
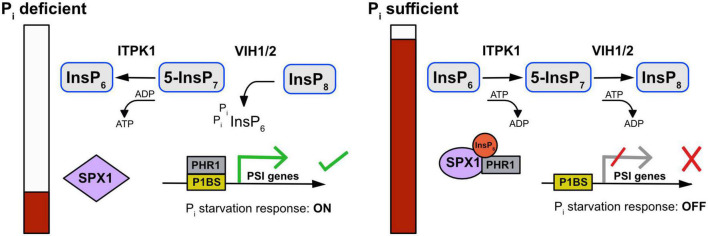
Model for the ITPK1-dependent phosphorylation of InsP6 and 5-InsP7 removal and possible link of ITPK1 with VIHs and phosphate homeostasis. Upon Pi-deficiency, ATP levels drop and stimulate ITPK1 to transfer the P-phosphate from 5-InsP7 to ADP, leading to the local generation of ATP and decreased 5-InsP7 levels. Additionally, low ATP/ADP ratios (i.e., low adenylate charge) and low Pi levels cause the switch from kinase to phosphatase activity of VIH proteins to hydrolyze InsP8. Lacking PP-InsPs, the interaction between PHR1 and SPX1 is destabilized, which promotes the binding of PHR1 to the P1BS motif in the promoter region of PSI genes. As a result, the Pi starvation response is activated. When plant cells regain sufficient Pi, ATP levels increase and the kinase activity of ITPK1 is activated, leading to the generation of 5-InsP7, which further serves as substrate for the kinase-activated VIH proteins to produce InsP8. Consequently, the accumulation of PP-InsPs facilitates the binding of SPX proteins to PHR1 to suppress Pi starvation responses.

This is explained by the severe PSR phenotype of the double mutant seedlings caused by the strong overaccumulation of P_i_, confirmed by the high expression of P_i_ starvation marker genes (Dong et al., 2019; Zhu et al., 2019). On the PP-InsP level, an increase in 5-InsP_7_ was observed in the double mutant, while InsP_8_ was below the limit of detection (Zhu et al., 2019). In contrast, HPLC profiles of a *vih1 vih2* double mutant shown in Land et al. (2021) displayed reduced InsP_7_ and InsP_8_ levels. It is worth mentioning that the T-DNA insertion in this particular *vih2* allele (*vip1-2*) is positioned outside the core *VIH2* kinase domain. Taken together, the disruption of both *VIH1* and *VIH2* appears to result in the loss of the plant’s ability to maintain intracellular P_i_ levels due to defective InsP_8_ synthesis (Dong et al., 2019; Zhu et al., 2019). Notably, the *itpk1 vih2* double mutant displays inhibited plant growth and an increase of approximately 27% in shoot P levels (Riemer et al., 2021). This strongly suggests that the combined activities of ITPK1 and VIH2 are critical for maintaining P_i_ homeostasis in plants, by concomitantly generating both the precursor (5-InsP_7_) as well as the main substrate (InsP_8_) of P_i_ sensing (Figure 2; Riemer et al., 2021). Lack of a PSR phenotype of ITPK4-deficient plants that display reduced levels of InsP_6_, InsP_7_, and InsP_8_ (Kuo et al., 2018; Wang et al., 2021) suggests that regulation of phosphate homeostasis by InsP and PP-InsPs might be even more complex. Future work should try to clarify the identities of InsP_7_ and InsP_8_ isomers by CE-ESI-MS analyses and, by taking advantage of chiral selectors, to address the question which PP-InsP species are altered. Besides, whether certain InsP_7_ or InsP_8_ isomers, or even enantiomers, play antagonistic roles in regulating the interaction of free standing SPX proteins with PHR1/PHL1 still needs clarification.

Recent studies have pointed to a connection between plant’s P_i_ status and immune responses (Campos-Soriano et al., 2020; Val-Torregrosa et al., 2022). These findings are based on the involvement of a miRNA species (miR399) in the regulation of P_i_ homeostasis in Arabidopsis (Chiou et al., 2006; Paul et al., 2015). Upon P_i_ starvation, miR399 accumulates and represses its target gene *PHOSPHATE2* (*PHO2*, encoding an E2 ubiquitin-conjugating enzyme) that is responsible for phosphate transporter degradation, leading to an enhanced P_i_ uptake in plants (Fujii et al., 2005; Kraft et al., 2005; Chiou et al., 2006; Liu et al., 2012; Huang et al., 2016). In rice, miR399 overexpression resulted in P_i_ accumulation in leaves and higher susceptibility to the fungal pathogen *Magnaporthe oryzae*, which was also observed upon high P_i_ fertilization (Campos-Soriano et al., 2020). In contrast, Val-Torregrosa et al. (2022) demonstrated an enhanced resistance to necrotrophic and hemibiotrophic fungal pathogens in Arabidopsis lines overexpressing miR399, as well as in *pho2* loss-of-function lines. The high P_i_ accumulation in Arabidopsis leaves caused by *miR399* overexpression and lack of functional PHO2 was linked to an elevated ROS production. This was assumed to be related to an increased HR in these plants during pathogen infection. Besides the changes in ROS levels, the mutant lines showed elevated SA and JA levels, combined with the upregulation of SA- and JA-dependent defense genes (Val-Torregrosa et al., 2022). Intriguingly, *pho2* mutants were also shown to accumulate high levels of InsP_8_ (Riemer et al., 2021). As previously mentioned, InsP_8_ is a key player in JA- and P_i_ signaling (Laha et al., 2015, 2016; Dong et al., 2019; Riemer et al., 2021), raising the hypothesis that P_i_ homeostasis and pathogen defense mechanisms might be linked by the plant’s PP-InsP status.

Recently, Gulabani et al. (2021) demonstrated that the products of IPK1, ITPK1 and VIH2 kinase activities also function as crosstalk mediators between pathogen defense and P_i_ homeostasis, and that these enzymes act as suppressors of SA-dependent defense mechanisms. Strikingly, previous studies indicated the suppression of SA-responsive genes by PHR1 and as a consequence, *phr1 phl1* double mutants appear to be more resistant to infections with *PstDC3000* (Castrillo et al., 2017).

While *PR1* and *PR2* transcripts are upregulated in *ipk1*, *itpk1*, and *vih2* lines, which are compromised in InsP_8_ levels or disrupted in functional PSR, the opposite was observed with the introduction of *ipk1* and *itpk1* into the *phr1 phl1* mutant background (Gulabani et al., 2021). In this case, a reduced expression of both SA marker genes in comparison to Col-0 and *phr1 phl1* was observed (Gulabani et al., 2021). The authors assumed that the downregulation of SA-associated defense genes in PP-InsP-compromised mutants is stimulated by a PHR1/PHL1-dependent increase in PSR. Furthermore, it is known that PSI genes might harbor SA-inducible elements in their promoters (Baek et al., 2017). Double mutants of the SA-biosynthesis gene *SID2* in the *ipk1* and *itpk1* backgrounds, respectively, indeed resulted in decreased P_i_ overaccumulation phenotypes. Exogenous application of SA to wild-type plants led to increased transcripts of the PSI gene *SPX1* or the PAMP-responsive gene *WRKY38*, both shown to be reduced in the *ipk1 sid2* and *itpk1 sid2* mutants, strengthening the hypothesis that SA may directly activate the transcription of PSI-genes (Gulabani et al., 2021). The phenotypes observed in *ipk1*, *itpk1* and *vih2* mutants are assumed to be related to the low InsP_8_ concentration that was observed at least in *ipk1* and *vih2* lines (Gulabani et al., 2021; Riemer et al., 2021), supporting a further putative link between PP-InsP-driven PSR and the capability to defend against pathogens.

To summarize, the connection of PSR and pathogen defense might give another perspective of how P_i_ is managed in crops. By having a deeper understanding of the factors affecting P_i_ homeostasis in plants, a more precise adjustment of fertilizer conditions may be employed in the field. This might help, for instance, to avoid strong pathogen infestation caused by excessive application of P_i_, as well as to reduce environmental pollution and the depletion of global P_i_-deposits, all of which will improve sustainability in crop production.

### The role of inositol phosphates in auxin signaling

Auxin regulates a multitude of plant functions, including cell division, elongation, differentiation, embryonic development, root and stem tropisms, apical dominance, and flower formation (Young et al., 1990; Woodward and Bartel, 2005; Tanaka et al., 2006; Möller and Weijers, 2009; Leyser, 2010; Müller and Leyser, 2011; Christie and Murphy, 2013; Gallavotti, 2013; Geisler et al., 2014). This phytohormone coordinates those physiological processes by modulating the transcription of auxin-responsive genes through the action of the three protein families: TRANSPORT INHIBITOR RESPONSE1 (TIR1) and AUXIN- SIGNALING F-BOX proteins (AFB1-5), Aux/indole-3-acetic acid (IAA) transcriptional repressors, and the AUXIN RESPONSE FACTORS (ARFs) (Dharmasiri et al., 2005; Kepinski and Leyser, 2005; Tan et al., 2007). Auxin mediates their functions by binding to TIR1/AFB F-box proteins, enhancing the interaction of TIR1/AFB with Aux/IAAs repressors, which are in turn degraded by the Skp, Cullin, F-box-containing complex (SCF) ubiquitin ligase to activate ARF transcription factors (Wang and Estelle, 2014; Salehin et al., 2015). The Arabidopsis genome encodes 6 TIR1/AFBs, 29 Aux/IAA proteins, and 23 ARFs, which act combinatorically to regulate a wide range of auxin-dependent processes (Calderón Villalobos et al., 2012; Shimizu-Mitao and Kakimoto, 2014; Dinesh et al., 2016). The auxin co-receptors TIR1/AFB proteins comprise an F-box domain in the N terminus and 18 Leucine-rich repeat (LRR) domains at the C terminus (Tan et al., 2007). Structural analyses of the auxin co-receptor complexes purified from an insect cell line ectopically expressing Arabidopsis ASK1-TIR1 were instrumental in unveiling the molecular basis of auxin perception (Tan et al., 2007). The TIR1 crystal structure contained insect-derived InsP_6_ as a cofactor (Tan et al., 2007). InsP_6_ interacts with a highly basic surface area formed by 10 positively charged residues of TIR1, supporting the formation of the auxin binding pocket. These residues are also conserved in Arabidopsis AFBs, suggesting its binding importance in this subfamily of F-box proteins. When TIR1 is mutated in three residues that are involved in the coordination with InsP_6_, it fails to interact with either IAA7 or ASK1, implying a key role of InsP_6_ in the structural architecture of TIR1 (Calderón Villalobos et al., 2012). InsP_6_ interacts primarily with halves of the TIR1-LRR solenoids, loop-2, and the Arg403 residue. The Arg403 residue also binds to the carboxy group of auxin and is essential for the structural function of TIR1 (Calderón Villalobos et al., 2012). The authors demonstrated further that the mutation in His78, Arg403, and Ser438 residues of TIR1, which are involved in both auxin and InsP_6_ binding, failed to reconstitute the interaction between TIR1 and IAA7 in the presence of auxin (Calderón Villalobos et al., 2012). While these findings suggest an important function of InsP binding to TIR1, it needs to be investigated whether the designated InsP_6_ binding pocket of TIR1 can accommodate also other InsPs or is specific to InsP_6_. Even before InsP_6_ was identified as cofactor for the auxin receptor complex, inositol polyphosphates have been linked with several auxin-dependent physiological processes (Xu et al., 2005; Zhang et al., 2007). In Arabidopsis, two Inositol 1,4,5-Trisphosphate 3-Kinases (IPK2α and IPK2β) were found to harbor 6-/3-kinase activities and sequentially phosphorylate Ins(1,4,5)P_3_ to generate InsP_5_ [2-OH] *via* an Ins(1,3,4,6)P_4_ intermediate *in vitro* (Stevenson-Paulik et al., 2002; Xia et al., 2003). Expression analyses of *IPK2*α and *IPK2*β in different tissues of Arabidopsis plants pointed to a role of those kinases in plant growth and development (Xia et al., 2003; Xu et al., 2005). Silencing of *IPK2*α through antisense gene expression led to enhanced root growth and pollen germination in transgenic Arabidopsis plants (Xu et al., 2005), both of which are auxin-regulated processes (Fu and Harberd, 2003; Wu et al., 2008). Subsequent work investigating the physiological functions of IPK2β kinase uncovered that *IPK2*β is an early responsive gene that regulates axillary branching by an auxin signaling pathway. Furthermore, the application of exogenous IAA induced *IPK2*β expression and overexpressing of *IPK2*β results in altered auxin responses such as lateral root formation and primary root development (Zhang et al., 2007). Reverse Transcription-Polymerase chain reaction analysis (RT-PCR) of *IPK2*β overexpression lines revealed decreased expression of *CYP83B1*, a regulator of auxin production (Bartel et al., 2001; Woodward and Bartel, 2005; Zhang et al., 2007), and enhanced expression of *PIN4*, which mediates auxin transport (Friml, 2003; Zhang et al., 2007). Moreover, the expression levels of *MAX4* and *SPS*, which are required for auxin-mediated shoot branching, was downregulated in *IPK2*β overexpression lines (Tantikanjana et al., 2001; Sorefan et al., 2003; Bainbridge et al., 2005; Zhang et al., 2007). Future work on auxin responses using *ipk2*β knockout lines will provide more insight into the IPK2β functions in auxin signaling. *IPK2*α and *IPK2*β are homologous genes with high sequence similarities (Stevenson-Paulik et al., 2002), and deletion of a single gene might not reveal its biological function due to redundancy. To date, the generation of *ipk2*α *ipk2*β double mutants was not successful because the homozygous double knockout appears to be lethal probably due to defects in pollen development, pollen tube guidance, and embryogenesis (Zhan et al., 2015). As such, the catalytic dead variants of *IPK2*β could not complement *ipk2*α *ipk2*β–associated lethality, suggesting an essential role of inositol polyphosphate signaling in plant reproduction (Zhan et al., 2015).

Other InsP kinases were found to be also involved in auxin-dependent physiological processes. Notably, transcriptome analysis of *ipk1-1* plants showed that genes involved in root hair differentiation and root system development were misregulated in the mutant line. Moreover, *ipk1-1* plants display a phenotype similar to the *mrp5* mutant (Kuo et al., 2014). *MRP5* encodes an ABC-type transporter mediating InsP_6_ loading into the vacuole (Nagy et al., 2009). In consequence, *mrp5* mutant plants display reduced levels of InsP_6_, as well as elevated cytoplasmic levels of InsP_7_ and InsP_8_ (Desai et al., 2014; Laha et al., 2019), and exhibit a root system architecture (RSA) phenotype in response to elevated auxin (Gaedeke et al., 2001). Further, *ipk1-1* plants having reduced levels of InsP_6_ also exhibit an altered RSA, which might be caused by compromised auxin signaling (Kuo et al., 2014). In line with this, the *ipk1-1* mutant exhibited defects in gravitropic responses. Both *ipk1-1* and *mrp5* mutant plants were also insensitive to exogenous auxin supply, as evidenced by an increase in relative primary root length (Gaedeke et al., 2001; Laha et al., 2020). Taken together, these findings put forward the importance of IPK1 function in auxin signaling. The fact that the *mrp5* mutant has elevated levels of InsP_7_ and InsP_8_ (Desai et al., 2014; Laha et al., 2019; Riemer et al., 2021), whereas *ipk1-1* is severely compromised in those PP-InsP species (Laha et al., 2015), raise the possibility that the decreased levels of InsP_6_ or PP-InsP might contribute to auxin signaling. To further corroborate the role of PP-InsPs in auxin responses, the *itpk1* and *vih2* mutant lines were investigated. The *itpk1* plants were shown to be defective in primary root elongation, leaf venation and compromised gravitropic root curvature, as well as thermomorphogenic adaptation, all of which are reminiscent of auxin deficient phenotypes (Laha et al., 2020). In auxin sensitivity assays, *itpk1* plants displayed resistance to exogenous auxin, which could be fully rescued by *itpk1* lines carrying a genomic *ITPK1* fragment. This reinforces the idea that phenotypic defects of *itpk1* mutant lines might be related to impaired auxin perception (Laha et al., 2020). ITPK1-deficient plants are defective not only in 5-InsP_7_ synthesis but are also perturbed in lower inositol phosphates homeostasis (Laha et al., 2019, 2020; Riemer et al., 2021), and their role in building auxin receptor complexes cannot be ignored (Laha et al., 2020). Specifically, HPLC profiles of both *itpk1* and *ipk1* mutants show reduced levels of InsP_5_ [1/3-OH], InsP_7_, and InsP_8_ and an increase in InsP_4a_, an unknown InsP_4_ isomer (Stevenson-Paulik et al., 2005; Laha et al., 2015).

Taken together, these results suggest that one or several inositol polyphosphate isomers might be important for auxin signaling. Future work is needed to clarify whether the control of auxin responses depends on the catalytic activity of ITPK1. Furthermore, the *vih2* mutant lacking detectable InsP_8_ levels, as revealed by SAX-HPLC, did not exhibit auxin-related phenotypes, suggesting that InsP_8_ might not be critical for auxin responses (Laha et al., 2020). To further clarify the role of InsP_8_ in auxin signaling, future work on *vih1 vih2* double knockout lines is necessary to account for a potential redundancy of the two VIH homologs.

Notably, competitive binding assays revealed that InsP_6_ and 5-InsP_7_ bind with similar affinities to the TIR1-ASK1-Aux/IAA7 auxin receptor complex (Laha et al., 2020). Considering the large amount of InsP_6_ present in plant cell extracts, an obvious question is how InsP_7_ (which comprises around 3% of global InsP_6_) could specifically control auxin perception. As mentioned earlier, several studies established that the major pool of InsP_6_ is stored in the vacuole (Nagy et al., 2009; Desai et al., 2014; Laha et al., 2019; Riemer et al., 2021), suggesting that the cyto/nucleo-plasmic concentration of InsP_6_ and InsP_7_ is distinct from the global cellular pool of InsP_6_ and InsP_7_. Investigating the localization of InsP_6_ and InsP_7_ at different compartments with the development of InsP_6_- and InsP_7_-specific sensors might clarify many of these open questions. Interestingly, a previous study reported that an InsP_6_ kinase interacts with certain protein complexes to generate InsP_7_ in close proximity to dedicated effector proteins (Rao et al., 2014). Similarly, recent work in Arabidopsis suggests that ITPK1 physically interacts with TIR1, presumably to channel 5-InsP_7_ to the auxin receptor complex (Laha et al., 2020). In addition, the potential of InsP molecules to induce a conformational change in TIR1 and promote the degradation of AUX/IAA is another conjecture to be solved. Knowing that different inositol phosphates have different affinities toward the auxin receptor complex is intriguing and raises the question whether these InsP molecules act differentially by forming distinct sets of auxin receptor complexes to regulate diverse auxin-related physiological processes. Altogether, many unsolved puzzles demand further research to identify the mechanism behind these phosphate-rich molecules playing a pivotal role in auxin-mediated plant growth and development.

In addition to the role of auxin in plant developmental and growth processes, several studies have also implicated a role of auxin in abiotic and biotic stresses (Cheong et al., 2002; Dowd et al., 2004; Hannah et al., 2005; Navarro et al., 2006; Wang et al., 2007; Jain and Khurana, 2009). The expression profiles of auxin-responsive genes of plants subjected to different biotic and abiotic stresses have pointed to a potential role of auxin in regulating plant defense responses, suggesting a possible crosstalk between auxin, abiotic and biotic stress signaling pathways (Ghanashyam and Jain, 2009). Recent findings revealed a potential role of auxin in regulating host-pathogen interaction. Auxin produced by different plant-associated microbes promotes disease susceptibility and antagonizes plant defense responses (Kunkel and Harper, 2018). Furthermore, *Arabidopsis thaliana* mutant lines defective in auxin signaling and perception showed increased levels of bacterial growth and suppressed host defenses, highlighting the role of auxin in biotic stress modulation (Djami-Tchatchou et al., 2020). Future work is needed to clarify whether inositol polyphosphates are involved in auxin-mediated pathogen defense responses.

## Outlook

**FIGURE 3:**
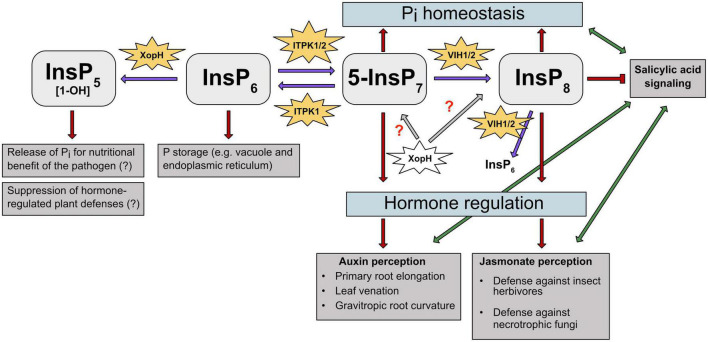
PP-InsPs and their kinases are involved in different abiotic and biotic stress responses in plants. PP-InsPs’ involvement in Pi homeostasis, hormone perception and regulation is depicted. Purple arrows indicate the kinase/phosphatase activity of the respective enzymes on InsPs and PP-InsPs. Gray arrows and red question marks depict a putative effect of XopH on PP-InsPs. Red arrows and T-shaped line indicate promotion and suppression of specific InsPs and PP-InsPs in regulating stress responses, respectively. Green arrows depict the interplay between plant hormones auxin, JA and SA, respectively. ITPK1 phosphorylates InsP6 to 5-InsP7. The latter serves as precursor for InsP8, which plays a crucial role in adaption to changing Pi levels. Additionally, InsP6 is degraded by XopH to potentially release Pi for the pathogen’s nutritional benefit. ITPK1 and VIH2 interaction is needed to maintain Pi homeostasis. Higher PP-InsPs are also involved in hormone perception and regulation. The ITPK1-generated 5-InsP7 is speculated to be involved in auxin and SA signaling. The VIH2- generated InsP8 has been proposed to represent a critical co-ligand of the JA receptor complex and is also assumed to regulate SA signaling. The bacterial type III effector XopH displays 1- phytase activity but may also have hydrolytic activities against PP-InsPs that might disrupt hormone-regulated defense mechanisms.

Here we pointed out the several roles InsPs and PP-InsPs play in regulating biotic and abiotic stress responses, and highlight these molecules as supporting modulators of plant metabolism to adapt to several environmental conditions (Figure 3). The recent development of more sensitive tools for the detection and quantification of low abundant PP-InsPs like CE-ESI-MS provides new insights into the large network of these molecules in eukaryotic systems (Qiu et al., 2020). However, the separation of enantiomeric PP-InsPs such as 1/3-InsP_7_ and 4/6-InsP_7_ still remains challenging with current chromatographic and electrophoretic methods. Future research needs to develop methods to distinguish between the mirror images to delineate which isomers are relevant in living plants. Besides the identification of the enantiomeric identity of these PP-InsP species, it will be a milestone to determine the responsible kinase for the newly identified 4/6-InsP_7_ and to determine the physiological processes this isomer regulates. We speculate that this might involve also responses to biotic stresses. Furthermore, the involvement of PP-InsPs in hormone signaling still remains enigmatic. Besides the role of these small molecules in auxin-, JA- and SA-dependent functions (Laha et al., 2015, 2020; Gulabani et al., 2021), a direct involvement in ethylene or brassinosteroid responses should be addressed, since a role of *myo-*inositol phosphate synthase in regulating plant growth and stress responses *via* ethylene- mediated signaling has been observed in Arabidopsis and wheat (Sharma et al., 2020a,b).

Finally, the identification of PP-InsPs and their different isomers will help to understand plant-pathogen interactions, which will be useful for improving crop growth and yield under abiotic and biotic stresses.

## Author contributions

ER and DL conceived and prepared the outline of this review. ER, NP, RY, PR, HJ, MK, GS, and DL wrote the manuscript. All authors contributed to the article and approved the submitted version.
